# Evaluation the possibility of vortex-induced resonance for a multistage pressure reducing valve

**DOI:** 10.1371/journal.pone.0266414

**Published:** 2022-04-01

**Authors:** Dongtao Xu, Changrong Ge, Ying Li, Yuejuan Liu

**Affiliations:** School of Mechanical Engineering & Automation, University of Science and Technology Liaoning, Liaoning Anshan, China; Tianjin University, CHINA

## Abstract

A multistage pressure reducing valve is presented in this paper. The pressure reducing components are specially designed to not only control the flow rate but also effectively prevent the cavitation vibration. However, when the fluid flows through the pressure reducing components, the divergence and shedding of the vortices in the flow field seriously affect the stability of the valve and cause vortex-induced vibration. Especially, the main frequency of the vortex shedding is in the same frequency range as the modal frequency of the valve, the vortex-induced resonance of the valve occurs. It seriously affects the safety of a control system. In this paper, by monitoring the lift coefficient of the vortex cross flow in the valve, the frequency spectrum information of the lift coefficient is used as the novelty indexes to indicate vortex-induced vibration of the fluid in the valve. The main frequency and amplitude of vortex-induced vibration are obtained. The factors affecting the vortex-induced vibration of the fluid are analyzed. The results indicate that vortex-induced vibration is the most serious when the valve is opened or closed. The variation of the flow velocity and the pressure difference have obvious effects on vortex-induced vibration of the valve. The intensity of the variation affects the main frequency and amplitude of vortex-induced vibration. Using thermal-fluid-solid coupling modal analysis instead of traditional modal analysis, the modal frequency under the working state of the valve is obtained. It is compared with the main frequency of vortex shedding, and vortex-induced resonance does not occur in the multistage pressure reducing valve.

## Introduction

In modern industrial production, the regulating valve is an indispensable component in a control system, as it can regulate the flow rate and stabilize fluid pressure [1–3]. With the development of science and technology, the requirements for the reliability and vibration characteristics of valves are increasing. To realize the functions of throttling and reducing pressure, the pressure reducing components of a valve are designed as labyrinth disc, window and porous sleeve types. Some scholars [4–6] have studied the combined structure of labyrinth discs and multistage sleeves to achieve pressure and noise reduction functions. In actual control systems with high pressure and other factors, when the fluid flows through the pressure reducing components, some vortices may form in the wake flow at the pressure reducing components. This vortex shedding is periodic and has a main frequency. When the main frequency of vortex shedding is equal to or close to the modal frequency of the valve, vortex-induced resonance will occur. This will cause large displacements and deformations in the valve trims, resulting in considerable vibrations and noise affecting the safety of the control system. Some scholars have carried out research on vortex-induced vibration phenomena. Wang Hai-min et al [7, 8] calculated the modal frequencies of a triple-eccentric butterfly valve and combined them with the frequency calculation formula of the Karman vortex street to determine conditions for no resonance. Kang Zhuang [9–12] studied the effects of hysteresis, surface roughness and other factors on the vortex-induced vibration of vertical pipes. Chizfahm A [13] studied the influence of wind speed on the lift coefficient of turbines and concluded that vortex shedding and structural oscillation were synchronized.

Vortex-induced vibration is a common problem in hydrodynamics. At present, most studies have only addressed the external flow field of the structure body. There are relatively few studies on the vortex-induced vibrations of the complex internal flow field in a regulating valve, and the damage caused by vortex-induced vibration is often one of the main failure forms in a control system. Therefore, research on the vortex-induced vibration of regulating valves has high value and significance.

A multistage pressure reducing valve is presented in this paper, and pressure reducing components are designed. This can effectively prevent the cavitation vibration in the valve caused by a sharp drop in fluid pressure. Using the turbulence model in Fluent software, the main frequency and amplitude of vortex shedding are obtained. The factors affecting the vortex-induced vibration of the fluid in the valve are analyzed. Using thermal-fluid-solid coupling modal analysis, the modal frequency of the valve is obtained. The possibility of vortex-induced resonance can be evaluated in the valve by comparing the two frequencies.

## Structural description of the multistage pressure reducing valve

**Fig 1:**
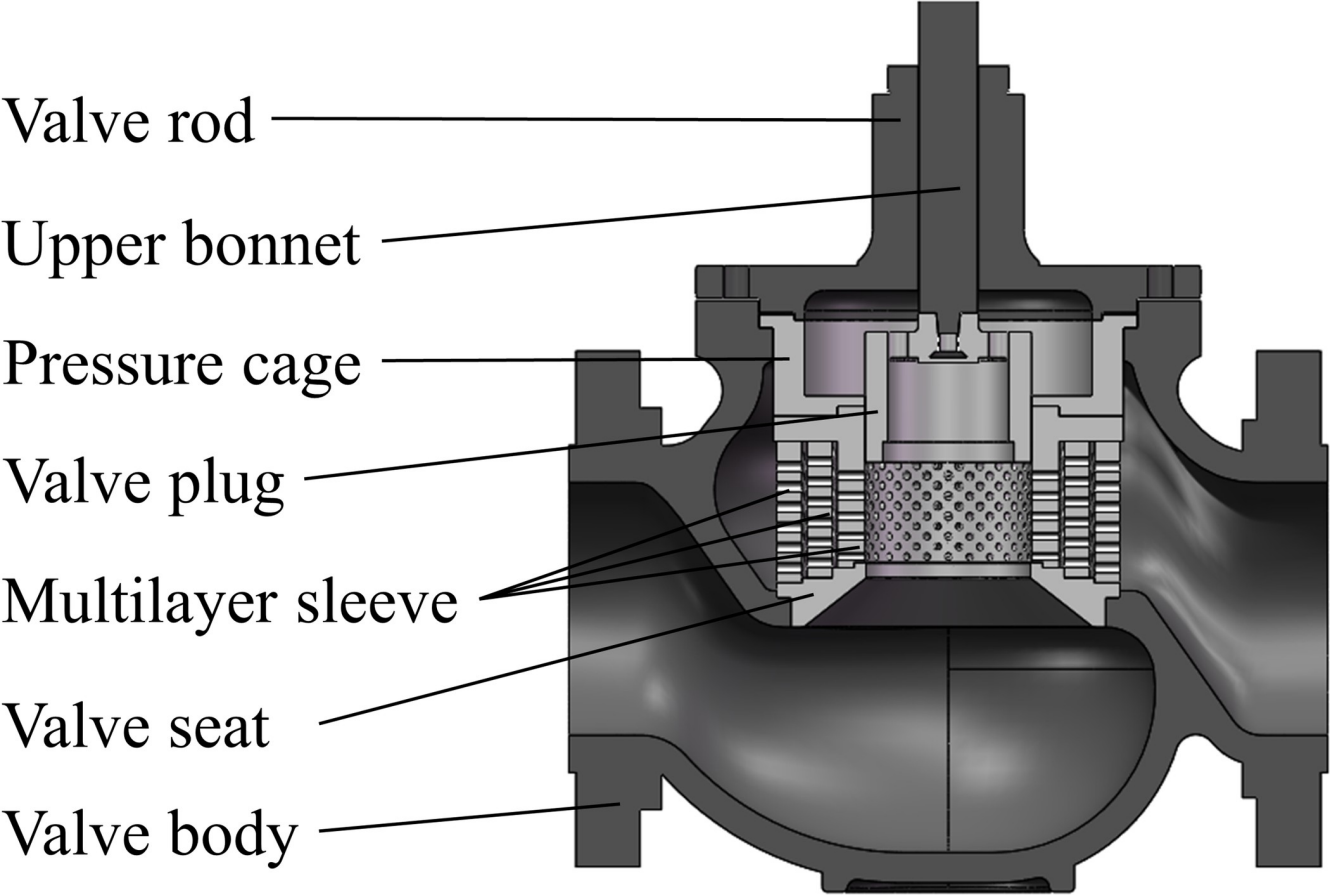
The structure of multistage pressure reducing valve.

The structure of the multistage pressure reducing valve is shown in Fig 1.

It consists of a valve body, valve seat, multilayer sleeve, valve plug, pressure cage, upper bonnet, and valve rod. The valve rod driven by the external actuator is able to drive the valve plug to move up and down. In this manner, the orifices on the inner sleeve of the multilayer sleeve are exposed to form effective flow area. The size and layout of the orifices directly affect the flow characteristics of the valve. The fluid in the valve is liquid water. When the fluid flows through the orifices, the static pressure of the fluid drops as the flow area decreases. If the fluid pressure suddenly drops to the saturated vapor pressure, cavitation vibration will occur. If the pressure difference of each stage pressure drop is greater than the blocked flow critical pressure difference, blocking flow will occur. The orifices of the multilayer sleeve can gradually reduce the pressure of the fluid in the valve to prevent cavitation vibration and blocking flow. The multilayer sleeve also has the advantages of easy processing and convenient maintenance.

## Orifice design of the multilayer sleeve

To avoid cavitation vibration and blocking flow in the valve, the throttle component is designed as a multilayer sleeve. The large pressure difference in the valve can be broken down into several small pressure differences. The pressure of the fluid will not drop sharply to the saturated vapor pressure. The structure of the multilayer sleeve is determined by the pressure difference at the inlet and outlet of the valve. If the number of decompression stages is too large, the structure will be complicated and difficult to realize [14, 15]. The number of decompression stages can be designed according to Eq (1):

$$n=\frac{p_{1}‐p_{2}}{2.5\times 10^{6}}$$ (1)

where *p*_1_ and *p*_2_ are the fluid pressures at the inlet and outlet of the valve, respectively.

The pressure difference of the blocked flow can be expressed as:

$$\Delta p\prime =F_{L}^{2}(p_{1}‐F_{F}p_{V})$$ (2)

where *F*_*L*_ is the pressure recovery coefficient of the liquid; *F*_*F*_ is the critical pressure ratio coefficient of the liquid, $F_{F}=0.96‐0.28\sqrt{p_{V}/p_{C}}$; *p*_*V*_ is the saturated vapor pressure; and *p*_*C*_ is the thermodynamic critical pressure.

According to the principle of multistage pressure drop, the pressure drop of each stage reduces in a geometric progression:

Δp=Δp1+Δp2+Δp3+…+Δpn=Δp1+Δp12+Δp122+…+Δp12n−1 (3)

where Δ*p* is the total fluid pressure difference; Δ*p*_*i*_ is the *i*-stage pressure drop (*i* = 1, 2, …*n*); and *n* is the theoretical number of decompression stages.

Considering the two factors of cavitation vibration and noise comprehensively, the pressure difference of each stage can be expressed as:

$$\Delta p_{i}=\Delta p(\frac{2^{n-i-1}}{2^{n}-1}+\frac{1}{2n})$$ (4)

After the pressure difference of each stage is determined, the throttle area on the middle and outer sleeves can be calculated according to the Bernoulli equation of energy. The flow area of the orifices is obtained as follows:

$$A_{e}=Q_{\max}\sqrt{\xi }\sqrt{\frac{\rho }{2\Delta p}}$$ (5)

where *Q*_max_ is the volume flow rate at a 100% opening, *ρ* is the density of the fluid, and *ξ* is the flow resistance coefficient.

According to Eq (5), the flow area can be obtained at a 100% opening. The design of the outer and middle sleeves can be designed by allocating the same small holes. The inner sleeve not only has the function of reducing pressure but can also control the flow rate of the valves at each opening, so that the designed valve can satisfy the required flow characteristics. The flow characteristic of the valve is the ratio between the relative flow rate and the relative stroke of the valve. The linear flow characteristic satisfies the proportional relationship between the relative flow rate and the relative stroke. The change of the flow rate caused by the change of unit stroke is constant. The functional relationship between the two can be described as:

$$\frac{Q}{Q_{\max}}=\frac{R-1}{R}\cdot \frac{l}{L}+\frac{1}{R}$$ (6)

where *q* is the relative flow rate; *Q* is the flow rate at a certain opening; *Q*_max_ is the flow rate at a 100% opening; *l* is the valve plug position at a certain opening; *L* is the stroke; *R* is the adjustable ratio (1/50).

**Fig 2:**
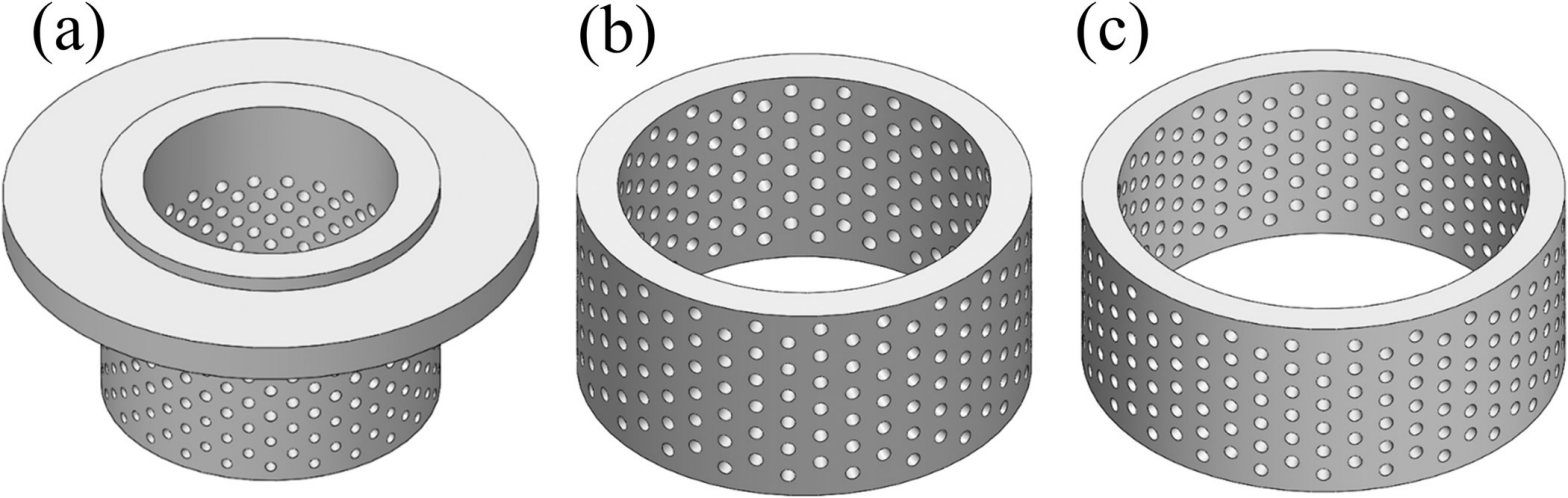
Multilayer sleeve. (a) inner sleeve. (b) middle sleeve. (c) outer sleeve.

When the nominal diameter of the valve is 250 mm, the inlet and outlet working pressures are 10 and 3 MPa, respectively, and the valve is designed according to the linear flow characteristics; the circulation volume (*Cv*) of the valve is 360 m^3^/h; the multilayer sleeve is designed as shown in Fig 2.

## Flow field simulation of the multistage pressure reducing valve

### Establishment of the simulation model

Before simulation, the 3D model of the valve is established, and the fluid model corresponding to each opening is generated by reverse modeling. The nominal diameter of the valve is Φ250 mm, and the valve plug diameter is Φ165 mm. According to the actual working conditions, the fluid in the valve is liquid water. The working pressure at the inlet of the valve is 10 MPa, the working pressure at the outlet is 3 MPa, and the fluid temperature is 473.15 K. The standard wall function is used and the gravity acceleration is considered in the flow field. The RNG *k-ε* turbulence model and the coupled algorithm are applied to the simulations to ensure computational accuracy [16, 17].

To simulate the flow field of the valve, the fluid model is divided into tetrahedral and hexahedron hybrid meshes using the ANSYS meshing tool. The grid independence is checked, and the reference value of grid independence is based on the flow rate and the average flow velocity at the outlet of the valve at a 100% opening. The test data is shown in Table 1:

**Table 1:** Fluid grid independence test data.

Number of grid cells	Outlet velocity (m/s)	Outlet flow (m^3^/h)
3951741	15.698	2772.558
4263695	15.709	2773.955
5177818	15.805	2789.258
6361212	15.811	2788.952

**Fig 3:**
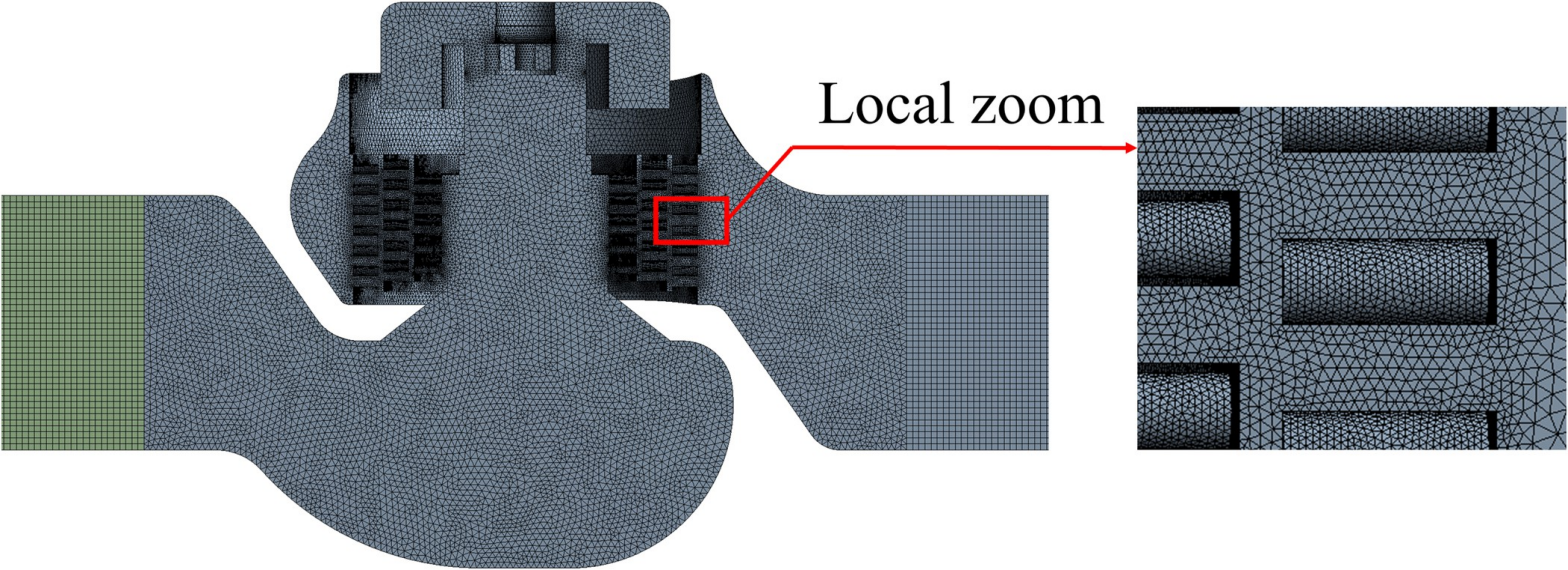
Fluid mesh model of the multistage pressure reducing valve.

When the number of grids increases from 5177818 to 6361212, the outlet velocity increases by 0.038% and the outlet flow decreases by 0.01%. When the number of grid cells is more than 5177818, the change in velocity and flow rate at the outlet can be ignored. Further refinement of the finite element mesh will not significantly affect the simulation results. Fig 3. shows the fluid mesh model.

### Numerical simulation of the flow field

**Fig 4:**
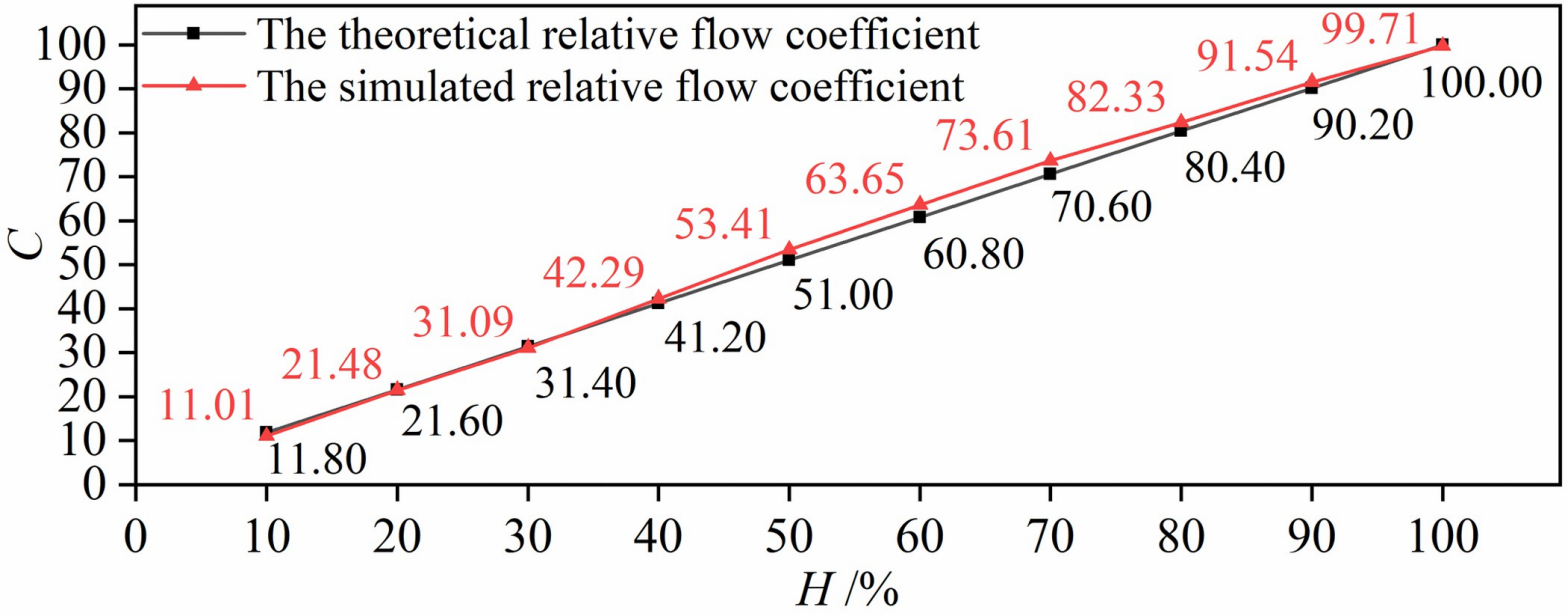
Comparison of the simulated relative flow coefficient with theoretical values.

The stroke of the valve plug is 100 mm. The volume flow rate at the outlet of the valve is monitored. After the iterative calculation, the circulation volume (*Cv*) of the valve is 358.96 m^3^/h. *C* is the relative flow coefficient and is dimensionless; it is the ratio of the flow rate at a certain opening to the circulation volume (*Cv*). According to Eq (6), when the adjustable ratio is 1/50, the theoretical relative flow coefficient at each opening is calculated. The relative flow coefficients obtained by simulation are compared with the theoretical relative flow coefficients, as shown in Fig 4.

Fig 4 shows that the simulated relative flow coefficient at each opening is within the allowable error range. The maximum error is at a 10% opening, and the error value is only 6.69%. This shows that the valve has a good linear flow characteristic.

**Fig 5:**
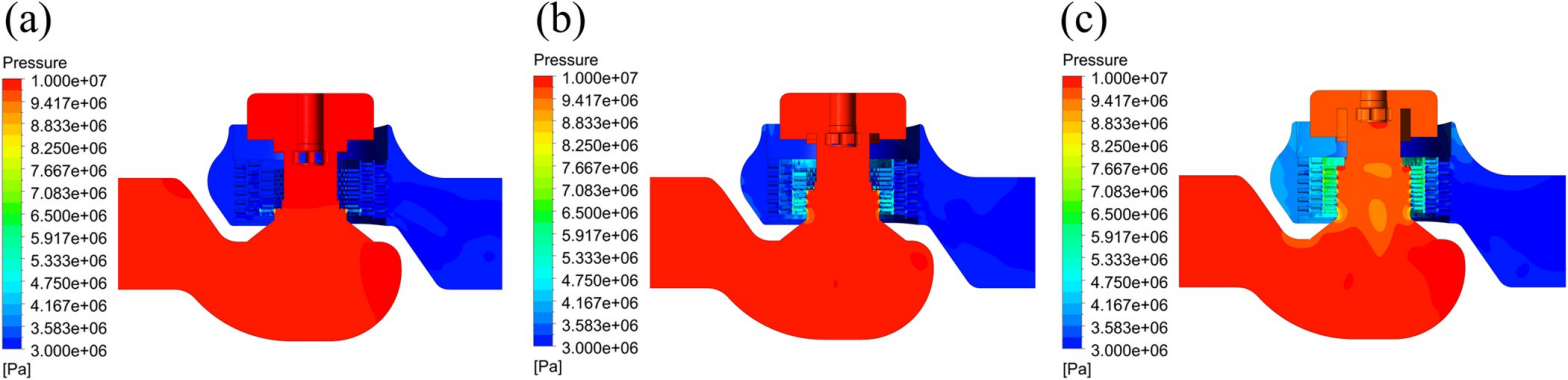
Pressure distributions of the flow field at different openings. (a) 10% opening. (b) 50% opening. (c) 100% opening.

**Fig 6:**
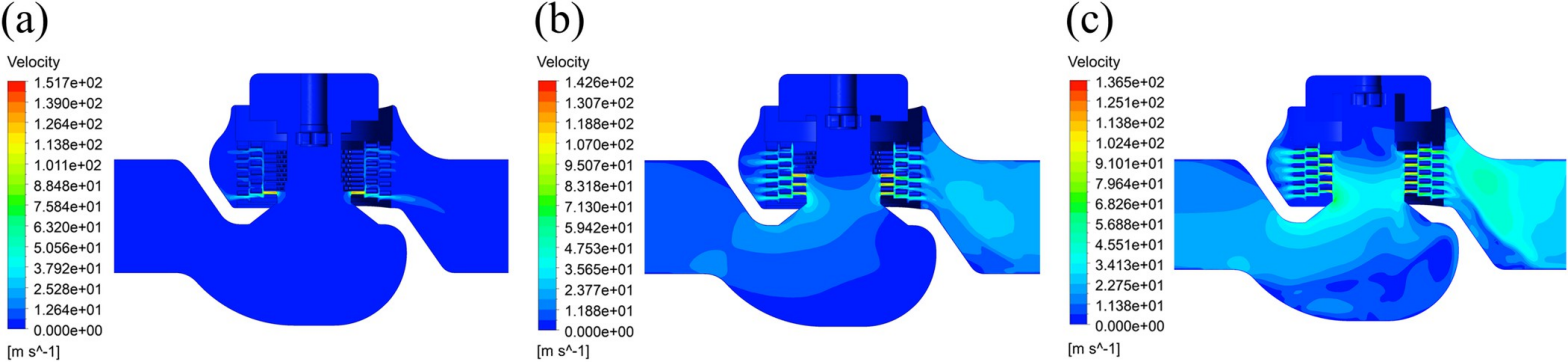
Velocity distributions of the flow field at different openings. (a) 10% opening. (b) 50% opening. (c) 100% opening.

The steady-state field under the same conditions is taken as the initial value of the transient field to accelerate the convergence. The computational domain is the whole fluid field. The time step size is set to 0.00025 s, the number of time steps is set to 8000, and the pressure distribution and the velocity distribution of the flow field at different openings are obtained at the 2nd second, as shown in Figs 5 and 6.

Fig 5 shows the pressure distributions of the flow field at openings of 10%, 50%, and 100%, respectively. The maximum and minimum pressures are 10 MPa and 3 MPa, respectively, and they are distributed at the inlet and outlet of the valve. At a 10% opening, only some orifices in the lower part of the inner sleeve are in the flow state. The fluid pressure drops significantly when the fluid flows through the orifices. At this moment, the flow rate is relatively small, and the fluid pressure does not change significantly as the fluid flows through the middle sleeve and outer sleeve. At openings of 50% and 100%, when the fluid passes through the multilayer sleeve, the static pressure of the fluid drops significantly. The pressure distribution of the flow field at a 100% opening conforms to the designed pressure drop rule. The orifices of the inner sleeve not only control the flow rate well but also have a pressure reducing function. The pressure reduction capacity of the valve is proportional to the opening.

Fig 6 shows the velocity distributions of the flow field at openings of 10%, 50%, and 100%. In each opening, the maximum velocity of the fluid is distributed at the orifices. At a 10% opening, the fluid velocity is the largest, at 151.7 m/s. The maximum velocity of the fluid in the valve is inversely proportional to the opening, and the velocity at the outlet of the valve is proportional to the opening.

**Fig 7:**
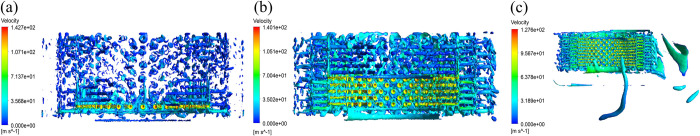
Velocity distributions of the vortex core at different openings. (a) 10% opening. (b) 50% opening. (c) 100% opening.

The velocity distributions of the vortex core at different openings at the 2nd second is obtained, as shown in Fig 7.

Most vortex cores are distributed at the orifices of the multilayer sleeve. the number of vortices at the orifices is proportional to the opening and the vortex velocity is inversely proportional to the opening. The large vortices gradually turn into small vortices, and some vortices begin to shed, which can easily cause vortex-induced vibration of the fluid in the valve.

## Analysis of vortex-induced vibration characteristics based on the lift coefficient

When vortex shedding occurs, a drag force is formed in the downstream direction, and a lift force is formed in the cross-flow direction. The lift coefficient represents the average lift force and is dimensionless.

The peak frequency of the lift coefficient amplitude is the main frequency of vortex shedding. The vortex shedding frequency can be obtained by fast Fourier transform (FFT) on the time-domain information of the lift coefficient obtained by the simulation of the transient flow field [18]. The lift coefficient expression is [4]:

$$C_{L}=\frac{F}{0.5\rho U^{2}S}$$ (7)

where *F* is the vortex-induced lift force, N; *ρ* is the density of the fluid, kg/m^3^; *U* is the fluid velocity in the vortex core, m/s; *S* is the windward area, m^2^.

**Fig 8:**
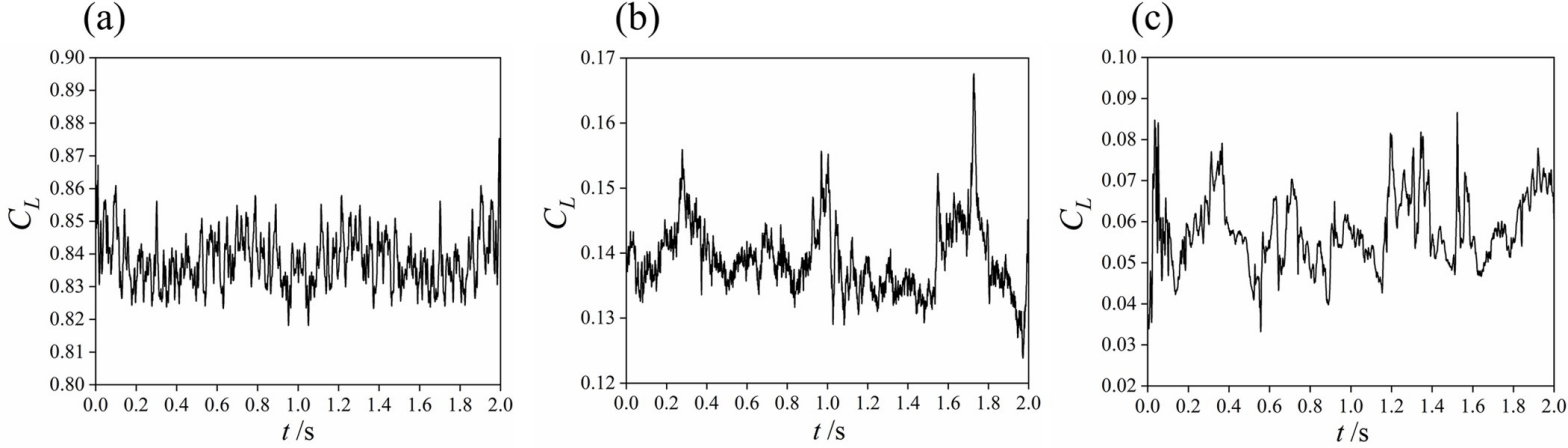
Time domain curves of the lift coefficient in 2 s. (a) 10% opening. (b) 50% opening. (c) 100% opening.

In the transient flow field, the calculation domain is the entire flow field. The lift coefficients of the vortices at different openings are monitored, and the corresponding time-domain information is obtained. The time-domain information of the lift coefficients for 3 typical openings are shown in Fig 8, and the range of lift coefficient values at different openings are shown in Table 2.

**Table 2:** The extreme values of the lift coefficient at different openings.

Opening /%	10	30	50	70	90	100
Maximum value	0.876	0.206	0.124	0.103	0.058	0.087
Minimum value	0.818	0.238	0.168	0.190	0.109	0.033

The maximum value of the lift coefficients is 0.876, at a 10% opening. The minimum value of the lift coefficients is 0.033, at a 100% opening. The variation range of the lift coefficient at each opening is relatively small. The value of the lift coefficient gradually decreases as the opening increases.

**Fig 9:**
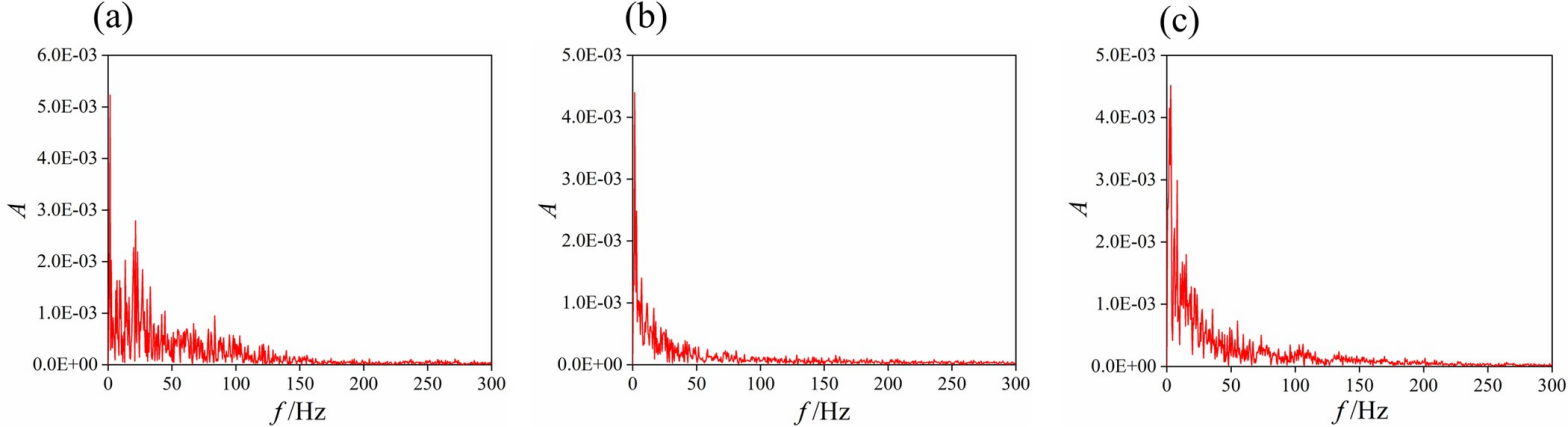
Frequency spectrum of the lift coefficient. (a) 10% opening. (b) 50% opening. (c) 100% opening.

The fast Fourier transform (FFT) is applied to the time-domain information of the lift coefficient, and the frequency-domain curve of the lift coefficient for the 3 typical openings are shown in Fig 9 and the frequency-domain characteristics for the other openings are shown in Table 3.

**Table 3:** Frequency domain characteristics of the lift coefficient.

Opening	10%	30%	50%	70%	90%	100%
*f* /Hz	0~150	0~100	0~50	0~50	0~50	0~50
*A*_max_	5.23E-3	4.24E-3	4.4E-3	4.5E-3	4.51E-3	4.52E-3

In Fig 9, the ordinate is the amplitude of the lift coefficient, the abscissa is the frequency of the vortex shedding. The main frequency of vortex shedding is the peak frequency of the amplitude. At a 10% opening, the peak frequency of the amplitude is in the range of 0~150 Hz, that is, the main frequency of vortex shedding is within 150 Hz. The maximum value of the amplitude is 5.23E-3. At a 30% opening, the main frequency of vortex shedding is within 100 Hz and the maximum value of the amplitude is 4.24E-3. At the other opening, the main frequency of vortex shedding is within 50 Hz and the maximum value of amplitude is 4.52E-3.

Obviously, at a 10% opening, the amplitude of vortex-induced vibration is the largest and the main frequency range of vibration is the widest. As the opening increases, the frequency range of vortex-induced vibration gradually decreases, the amplitude of vortex-induced vibration decreases firstly, and then increase slightly. Therefore, vortex-induced vibration is the most serious when the valve is opened and closed.

According to the simulation analysis of the flow field, although the inlet and outlet pressure of the valve is constant, the throttling area changes when the opening changes, so the fluid pressure and velocity near orifices changes. The variation of the flow velocity and pressure difference at the multilayer sleeve have obvious influence on vortex-induced vibration of the valve. The intensity of the variation affects the degree of vortex-induced vibration. Although a large number of vortices appear at the throttle holes in the multilayer sleeve, the multilayer sleeve can effectively reduce the amplitude and frequency of vortex-induced vibration.

## Evaluation the possibility of vortex-induced resonance

Under the ANSYS Workbench platform, the fluid module, temperature field module, static module and modal analysis module are combined to calculate the prestress mode of the valve. In the fluid module, the pressure and temperature distribution can be obtained by calculating the steady-state flow field of the valve; in the temperature module, the temperature distribution of the valve is obtained by transmitting the fluid temperature to the valve through the coupling surface; in the statics module, the fluid pressure is transmitted to the valve through the coupling surface, and the temperature of the valve in the temperature field is imported into the statics module; then, the calculation information of the statics module is imported into the modal module to complete the modal calculation.

Traditional modal analysis (dry mode) only considers the valve system’s own factors, such as shape and boundary conditions. In this paper, multiphysics coupled modal analysis (wet mode) is applied. The fluid pressure is the external excitation force, and the temperature is the internal excitation force, so the modal frequency of the structure is more accurate [19–21].

### Solid mesh generation and material parameters

**Fig 10:**
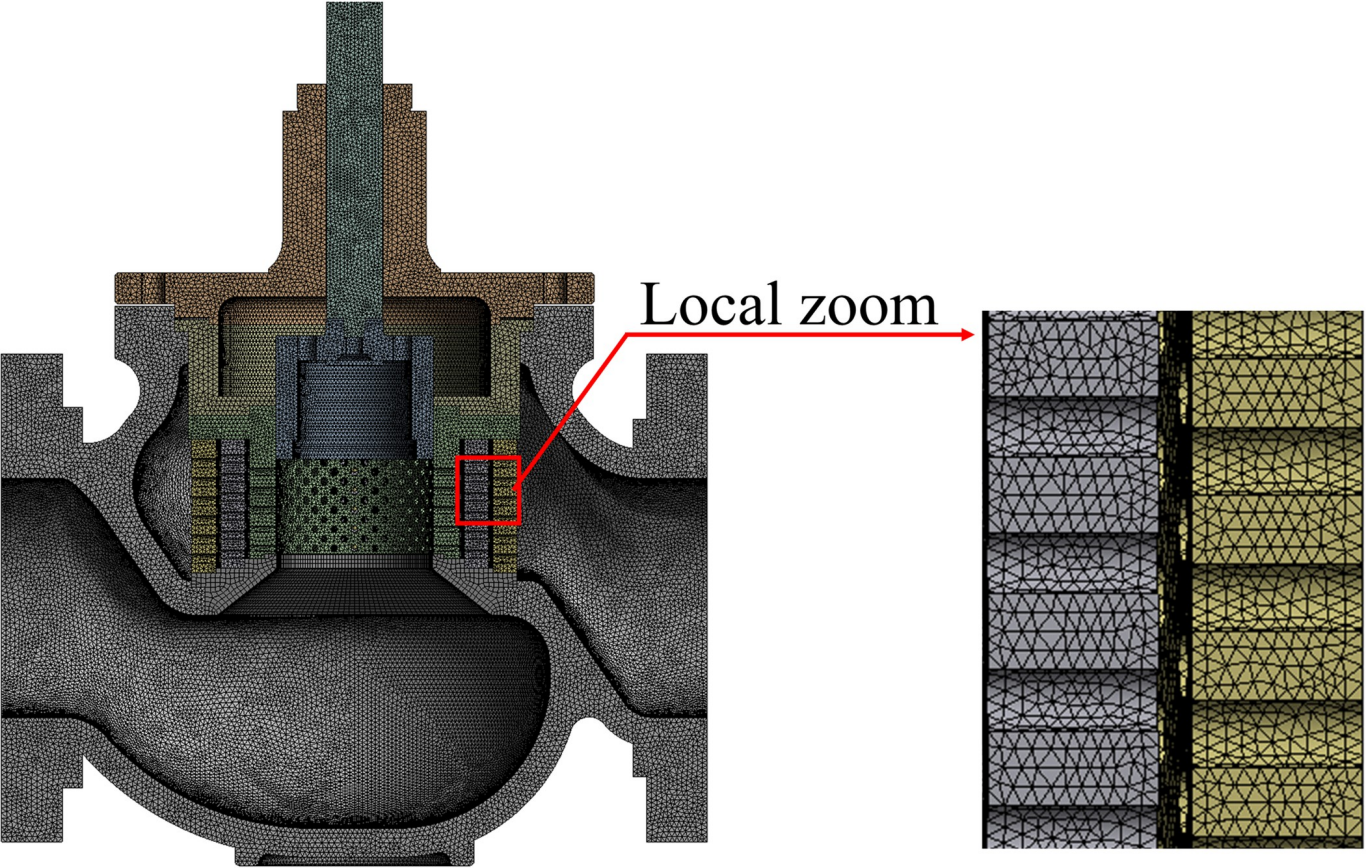
Solid finite element mesh of the valve at a 100% opening.

Using ANSYS meshing software, the valve is divided into tetrahedral and hexahedral hybrid meshes. The structure of the orifices is partially refined. Taking the mode at a 100% opening as an example, its mesh is shown in Fig 10.

## Thermal-fluid-solid coupling modal analysis and evaluation the possibility of vortex-induced resonance

In the statics module, the pressure information and temperature information are loaded into the valve. A fixed constraint is applied to the inlet of the valve, and a displacement constraint is applied to the outlet. The influence of gravitational acceleration is taken into account. High-order modal frequencies can be considered combinations of several low-order modal frequencies. Therefore, a 1st- to 6th-order modal simulation analysis at different openings is carried out. Table 4 shows the material parameters of main parts. Table 5. shows the modal frequencies of each order of the valve.

**Table 4:** Material parameters of the main parts.

Parts	Material	Density /kg/m^3^	Modulus of elasticity /GPa	Poisson’s ratio	Thermal expansion coefficient /×10^−6^°C
Valve rod	316SST	7930	179	0.30	17.4
Valve seat					
Valve body	WCB	7750	202	0.30	11.55
Valve bonnet					
Valve plug	15CrMo	7880	212	0.28	13.37
Multilayer sleeve					
Pressure cage					

**Table 5:** Modal frequencies of the valve. /Hz.

Opening	1^st^ order	2^nd^ order	3^rd^ order	4^th^ order	5^th^ order	6^th^ order
10%	257.12	821.57	914.32	962.31	1226.10	1571.2
30%	257.04	819.20	914.21	962.28	1225.80	1570.20
50%	256.86	817.89	914.11	962.14	1224.30	1568.40
70%	256.79	816.23	912.36	958.80	1223.80	1566.40
90%	256.66	814.23	910.18	956.50	1223.50	1563.40
100%	255.96	812.58	908.03	951.95	1223.30	1560.60

The modal frequencies of each order are relatively smaller at large opening. At each opening, the high-order modal frequencies of the valve are greater. When the valve is at a 100% opening, the modal frequency of the valve is the smallest, which is 255.96 Hz. At the same opening, the modal frequency of the valve increases with the increase of modal order. At the same modal order, the modal frequency of the valve decreases with the increase of the opening.

When the main frequency of vortex shedding is the same or close to the modal frequency of a valve, vortex-induced resonance may occur. The modal frequency of the valve is greater than 255.96Hz. The main frequency of vortex-induced vibration is in the range of 0~150 Hz. By comparing the main frequency of vortex-induced vibration with the modal frequency of the valve, there is no equal or close frequency value at each opening, and vortex-induced resonance cannot occur in the valve.

## Conclusions

A multistage pressure reducing valve was studied in this paper. The multilayer sleeve was specially designed according to the linear flow characteristics, and vortex-induced vibration of fluid in the valve were studied. The conclusions were as follows:

1. The valve designed with the pressure reducing components not only satisfied the flow characteristics but can also effectively prevented cavitation vibration and blocking flow. When the fluid flowed through the pressure reducing components, the fluid pressure dropped step by step, the flow velocity increased rapidly, and a large number of vortices appeared near the orifices.
2. The frequency spectrum information of the lift coefficient was used as the novelty indexes to indicate vortex-induced vibration of the fluid. By monitoring the lift coefficient, the main frequency of vortex-induced vibration and amplitude of vortex-induced vibration were obtained, the main frequency was in the range of 0~150 Hz, the maximum amplitude was 5.23E-3. At a 10% opening, the amplitude of vortex-induced vibration was the largest and the main frequency range of vibration was the widest. Therefore, when the valve was opened and closed, vortex-induced vibration was the most serious. The variation of the flow velocity and the pressure difference had obvious influence on vortex-induced vibration of the valve. The intensity of the variation affected the degree of vortex-induced vibration. The pressure reducing components could effectively reduce the amplitude and frequency of vortex-induced vibration.
3. Combined with the flow field, temperature field, static field and mode analysis modules, the thermal-fluid-solid coupling modal frequency of the valve was analyzed. Based on wet modal analysis, the coupling influence of the temperature field on valve mode was specially added. At the same order state, the valve modal frequency was inversely proportional to the opening. The minimum value of the modal frequency in the operating state of the valve was 255.96 Hz. The main frequency of vortex-induced vibration was in the range of 0~150 Hz. There was no equal or close frequency value at each opening, the fluid had good vortex-induced characteristics, and vortex-induced resonance could not occur in the valve.

## Supporting information

S1 File Original data of tables and figures. (ZIP)
